# Similarity Studies of Corona Viruses through Chaos Game Representation

**DOI:** 10.4236/cmb.2020.103004

**Published:** 2020-09

**Authors:** Dipendra C. Sengupta, Matthew D. Hill, Kevin R. Benton, Hirendra N. Banerjee

**Affiliations:** 1 Department of Mathematics, Computer Science & Engineering Technology, Elizabeth City State University, Elizabeth City, North Carolina, USA; 2 Department Natural Sciences, Elizabeth City State University, Elizabeth City, North Carolina, USA

**Keywords:** Covid-19, Chaos Game Representation, Deoxyribonucleic Acid, Phylogenetic Analysis, Shannon Entropy

## Abstract

The novel coronavirus (SARS-COV-2) is generally referred to as Covid-19 virus has spread to 213 countries with nearly 7 million confirmed cases and nearly 400,000 deaths. Such major outbreaks demand classification and origin of the virus genomic sequence, for planning, containment, and treatment. Motivated by the above need, we report two alignment-free methods combing with CGR to perform clustering analysis and create a phylogenetic tree based on it. To each DNA sequence we associate a matrix then define distance between two DNA sequences to be the distance between their associated matrix. These methods are being used for phylogenetic analysis of coronavirus sequences. Our approach provides a powerful tool for analyzing and annotating genomes and their phylogenetic relationships. We also compare our tool to ClustalX algorithm which is one of the most popular alignment methods. Our alignment-free methods are shown to be capable of finding closest genetic relatives of coronaviruses.

## 1. Introduction

Deoxyribonucleic Acid (DNA) is a molecule that encodes the genetic instructions used in the development and functioning of all known living organisms. As such, DNA has become a subject of both theoretical and applied studies for the last decades. DNA is a polymer of nucleotides. Nucleotides are the building blocks of DNA. The four different nucleotides of DNA are: adenine (A), cytosine (C), guanine (G), and thymine (T).

DNA sequences analysis, as one of the most important parts of bioinformatics, which was considered to reveal the essence of all life phenomenon, has been developing rapidly in recent years. Sequence comparison is crucial to understand the evolutionary relationships among organisms. Many methods have been proposed to compare genetic sequences. Traditionally, most of these approaches are the widely used alignment-based methods. In these methods, molecular sequences are optimally aligned based on selected scoring systems. The alignment-based methods often give high accuracy and may reveal the relationships among sequences. Some algorithms have been established and incorporated into software for sequence alignments. However, one of the main drawbacks of these techniques is that they are very time-consuming and expensive in memory usage. As a result, alignment-free approaches such as in [1] [2] [3] have attracted more and more attention and have been applied to biological sequence comparison as well as phylogeny analysis.

Chaos Game Representation (CGR) is an iterative system method originally proposed by Jeffery [4]. CGR is a two-dimensional plot, where the primary sequence organization of DNA is mapping using iterative function. CGR patterns of DNA segments have been proposed as a method for the classification and Identification of genomic sequences [4]-[10]. The use of CGR has mostly been restricted to a visualization tool representing nucleotide sequences, in which patterns like over-or underrepresentation of nucleotides, dinucleotides, trinucleotides, etc. can be visually ascribed. Goldman concluded that the patterns exhibited by CGR are sufficient to evaluate word length composition of three, i.e., the frequencies of nucleotides, dinucleotides and trinucleotides [5]. However, it was shown later that longer oligonucleotide frequencies also influence the patterns seen in CGR [9]. Later, a spectrum of word lengths, in addition to nucleotide and dinucleotide, in CGRs were identified as factors that can differentiate between genomes of different species. Several distance measures were proposed to compare two or more CGRs and it was employed for studying phylogenetic relationships among diverse species [7] [8] [9] [10] [11]. However, it is not clear if intra-species genomic variability, which is much less than between-species variation, can be resolved using CGRs with similar word lengths. Later it was found in [12], the value k = 7 achieved the highest accuracy scores for HIV-1 subtypes classification.

The motivation of writing this paper came from the recent outbreak of novel coronavirus (SARS-Cov-2) now known as Covid-19. SARS-CoV-2 is the third pathogenic novel coronavirus to emerge over the past two decades. The first, discovered in 2003 and named SARS-CoV, caused SARS, a serious and atypical pneumonia. The second, MERS-CoV, emerged a decade later in the Middle East and caused a similar respiratory ailment called Middle East respiratory syndrome (MERS). Since its identification, 2494 cases of MERS-CoV infection and nearly 900 deaths have been documented [13]. The SARS-CoV epidemic proved larger but less deadly, with approximately 8000 cases and nearly 800 deaths [14]. There are other four coronaviruses that cause colds in humans—known as HCoV-229E, HCoV-NL63, HCoV-OC43 and HCoV-HKU1 [15].

In this paper, we proposed two methods, i.e., probability matrix method and centroid matrix method combining with CGR to construct distancematrix between two genomes, and then create dendrogram using Hierarchical Agglomerative Clustering (HAC) analysis. Our dendrogram can accurately identify the genetic relationship of different biology, and this method is generally applicable to various organisms.

## 2. Methods

In this section we first describe the dataset used for our analysis, then present an overview of the three main steps of the method and conclude with a description of the two distances that we considered.

### 2.1. Dataset

Data acquisition: All viral sequences downloaded in FASTA format from two databases for our analysis: NCBI (https://www.ncbi.nlm.nih.gov/) and GISAID (https://www.gisaid.org/).

For our experiment, we used only complete genomes of 15 corona viruses as it is given in Table 1.

### 2.2. Overview

The method we used to analyze and classify the 15 sequences of the dataset has three steps: 1) generate graphical representations (images) of each DNA sequence using CGR and define FCGR probability matrix and CGR centroid method using the features of CGR; 2) compute all pairwise distance to obtain two distance matrices; and 3) create the dendrogram of the distance matrix using Hierarchical Agglomerative Clustering (HAC) analysis.

**Figure 1.**
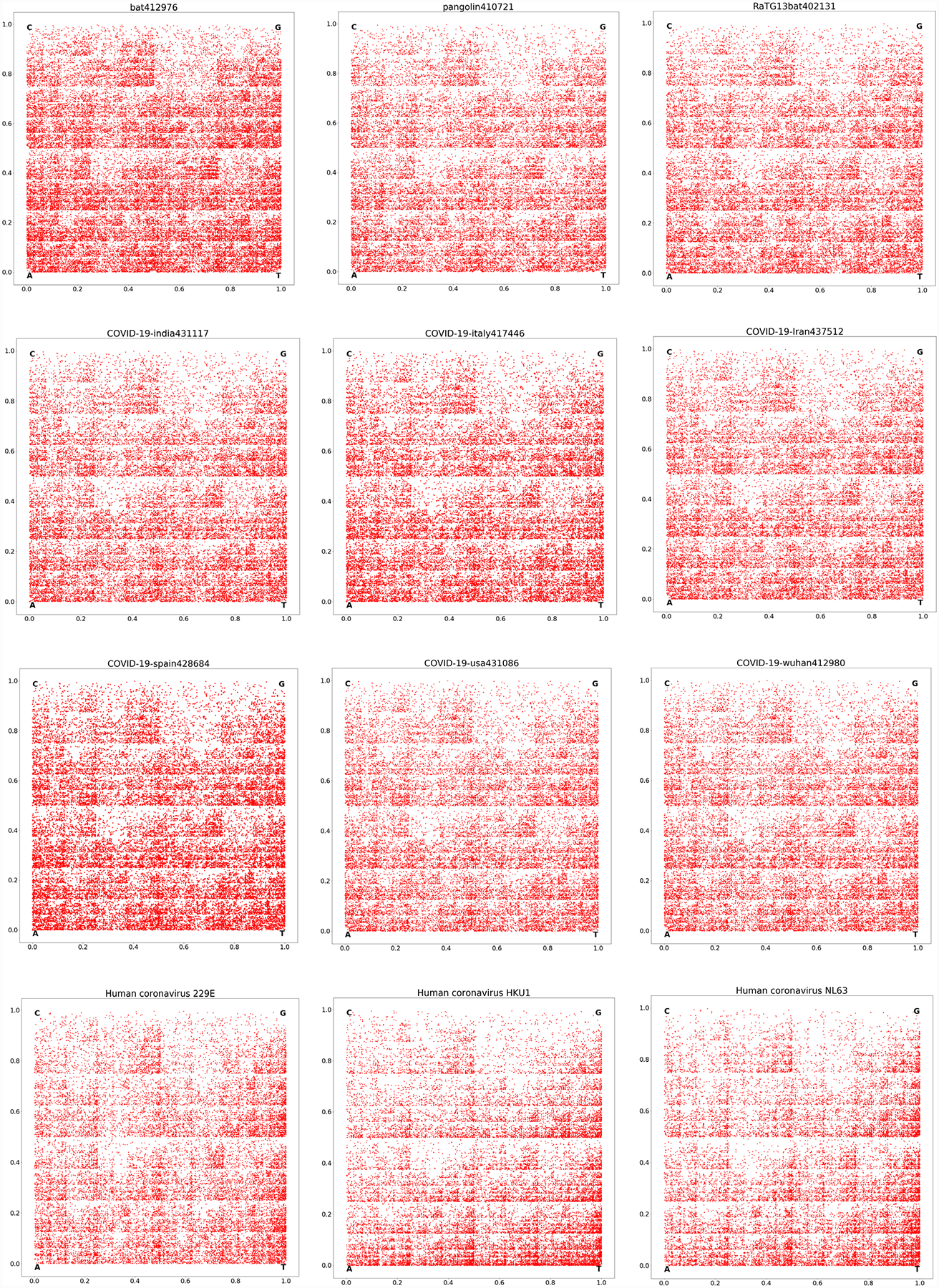

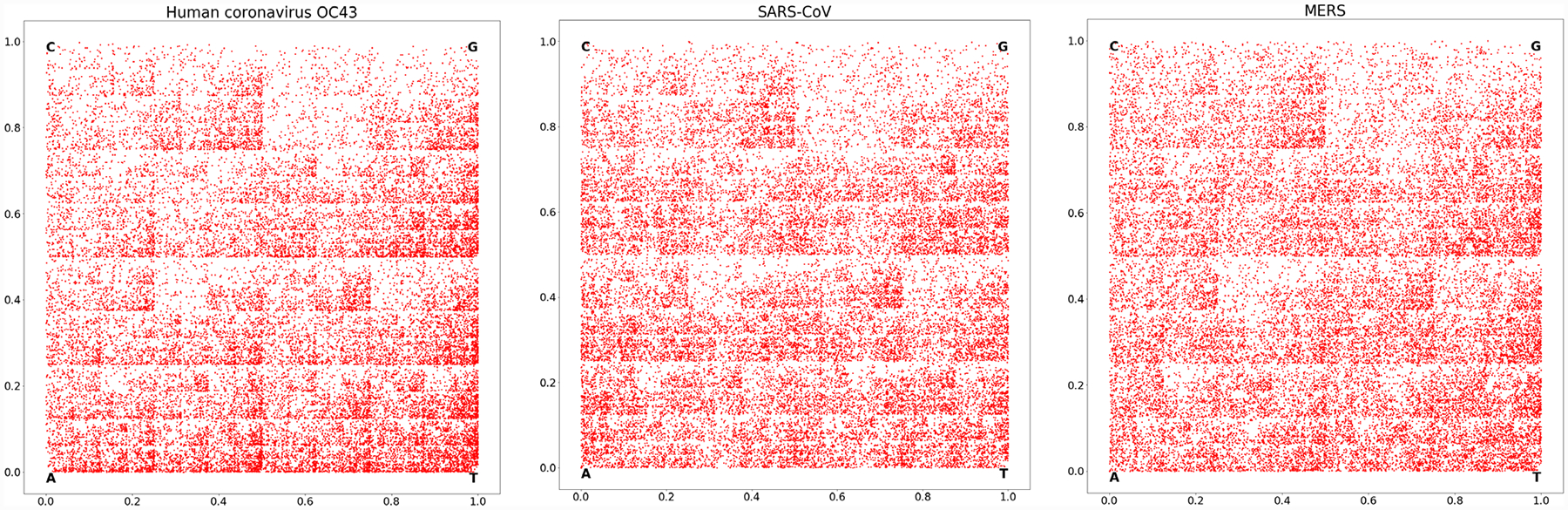
CGR images of all fifteen coronaviruses listed in Table 1.

CGR is an iterative method introduced by Jeffery [4] to visualize the structure of a DNA sequence. A CGR associates an image to each DNA sequence as follows: starting from a square with corner labeled four nucleotides C, G, A and T, and the center of the square as the starting point, the image is obtained by successively plotting nucleotide as the middle point between the current point and the corner labeled by the nucleotide to be plotted. If the generated square image has a size of 2^*k*^ × 2^*k*^ pixels, then every pixel represents a distinct k-mer: A pixel is color red if the k-mer it represents appears in the DNA sequence, otherwise it is white. CGR images of generating DNA sequences coming from various species show pattern such as squares, parallel lines, rectangles, triangles, and also complex fractal patterns. We have created CGR of all 15 virus genomes and visually they look similar (see Figure 1 below).

For step (1), we will use a slight modification version of the original CGR, introduced in [9]: a k-th order FCGR (Frequency Chaos Game Representation) is a 2^*k*^ × 2^*k*^ matrix that can be constructed by dividing the CGR plot into a 2^*k*^ × 2^*k*^ grid, and defining the element |*a*_*ij*_| is as the number of points that are situated in the corresponding grid square. A first-order FCGR and a second-order FCGR have the structure shown below, where *N*_*w*_ is the number of occurrences of the *k*-mer *w*, in the sequence *s* is

$$FCGR_{1}(s)=\left(\begin{array}{cc} N_{C} & N_{G}\\ N_{A} & N_{T} \end{array}\right)\text{ and }FCGR_{2}(s)=\left(\begin{array}{cccc} N_{CC} & N_{GC} & N_{CG} & N_{GG}\\ N_{AC} & N_{TC} & N_{AG} & N_{TG}\\ N_{CA} & N_{GA} & N_{CT} & N_{GT}\\ N_{AA} & N_{TA} & N_{AT} & N_{TT} \end{array}\right).$$

The (*k* + 1)th order *FCGR*_*k*+1_(*s*) can be obtained by replacing each element *N*_*X*_ in *FCGR*_*k*_ (*s*) with four elements $\left(\begin{array}{cc} N_{CX} & N_{GX}\\ N_{AX} & N_{TX} \end{array}\right)$ where *X* is a sequence of length *k* over the alphabet {A, C, G, T}. For each *k* ≥ 1, we can define a probability matrix of *FCGR*_*k*_ (*s*) by taking each entry of *FCGR*_*k*_ (*s*) dividing by the total counts of all *k*-mers. We denote the **FCGR probability matrix** by (*P*_*ij*_),1 ≤ *i, j* ≤ 2^*k*^. Note that $\underset{i,j}{\sum }P_{ij}=1$. Probability matrix can be interpreted as probability of distribution.

Since the CGR captures the information of the whole genome data, extracting the global features from the CGR may not be efficient enough to distinguish the genomes. In **CGR Centroid method**, we concentrate on extracting the local features as shown in [11]. We partition the CGR into sub-regions so that it reveals local information of the interested areas. If two dots are within the same quadrant, they correspond to sequences with the same last mononucleotide; if they are in the same sub-quadrant, the sequences have the same last dinucleotides; and so on. This can demonstrate the structure of the sequences yielding the points in the CGR. Chaos Centroid method utilizes this biological significance by computing the centroid of the distributed points of each sub-region.

For Chaos Centroid method, the CGR is partitioned into 10×10 equal subregion. The choice of 10 is to minimize the computation time. For each partition, we compute the centroidas follows. Let (*x*_*k*_, *y*_*k*_) be the coordinates of a point in the CGR. We define the centroid in each of the 10×10 grid as follows:

$$c_{ij}=\left(\frac{\overset{\left|a_{ij}\right|}{\underset{k=1}{\sum }}x_{k}}{\left|a_{ij}\right|},\frac{\overset{\left|a_{ij}\right|}{\underset{k=1}{\sum }}y_{k}}{\left|a_{ij}\right|}\right),1\leq i,j\leq 10.$$

For step (2), after computing FCGR probability matrices and computing centroid for each of the sequences in the dataset, the goal was to measure “distance” between two CGR images. There are many distances as it is given in [10] [11] that can be defined for our purpose. One of the goals of this study was to identify what distance is better able to differentiate the structural differences of various genomic DNA sequences. In this paper we use two different distances: FCGR Probability Matrix distance and CGR Centroid distance. Both use the Euclidean distance. For step (3), after computing all pairwise distances we obtained two different distance matrices. Then, we created the dendrogram of the distance matrices using Hierarchical Agglomerative Clustering (HAC) analysis.

### 2.3. Distances

In this section we formally define each of the two distances. For two FCGR probability matrices (*p*_*ij*_) and (${p^{\prime }}_{ij}$) we define $d_{ij}=\left|p_{ij}-{p^{\prime }}_{ij}\right|$ The distance between the two probability matrices denoted by $D_{PM}=\sum_{i=1}^{2^{k}}\sum_{j=1}^{2^{k}}d_{ij}$ For two genomes, we calculate 100 centroids *c*_*ij*_ = (*x*_*ij*_*, y*_*ij*_) and ${c^{\prime }}_{ij}=({x^{\prime }}_{ij},{y^{\prime }}_{ij})$ respectively for 1 ≤ *i*, *j* ≤ 10. Then we found Euclidean distance between them $d_{ij}=\sqrt{{\left(x_{ij}-{x^{\prime }}_{ij}\right)}^{2}+{\left(y_{ij}-{y^{\prime }}_{ij}\right)}^{2}}$. Then calculated the centroid distance between two genomes denoted by $D_{cd}=\sum_{i=1}^{10}\sum_{j=1}^{10}d_{ij}$.

## 3. Results

**Figure 2.**
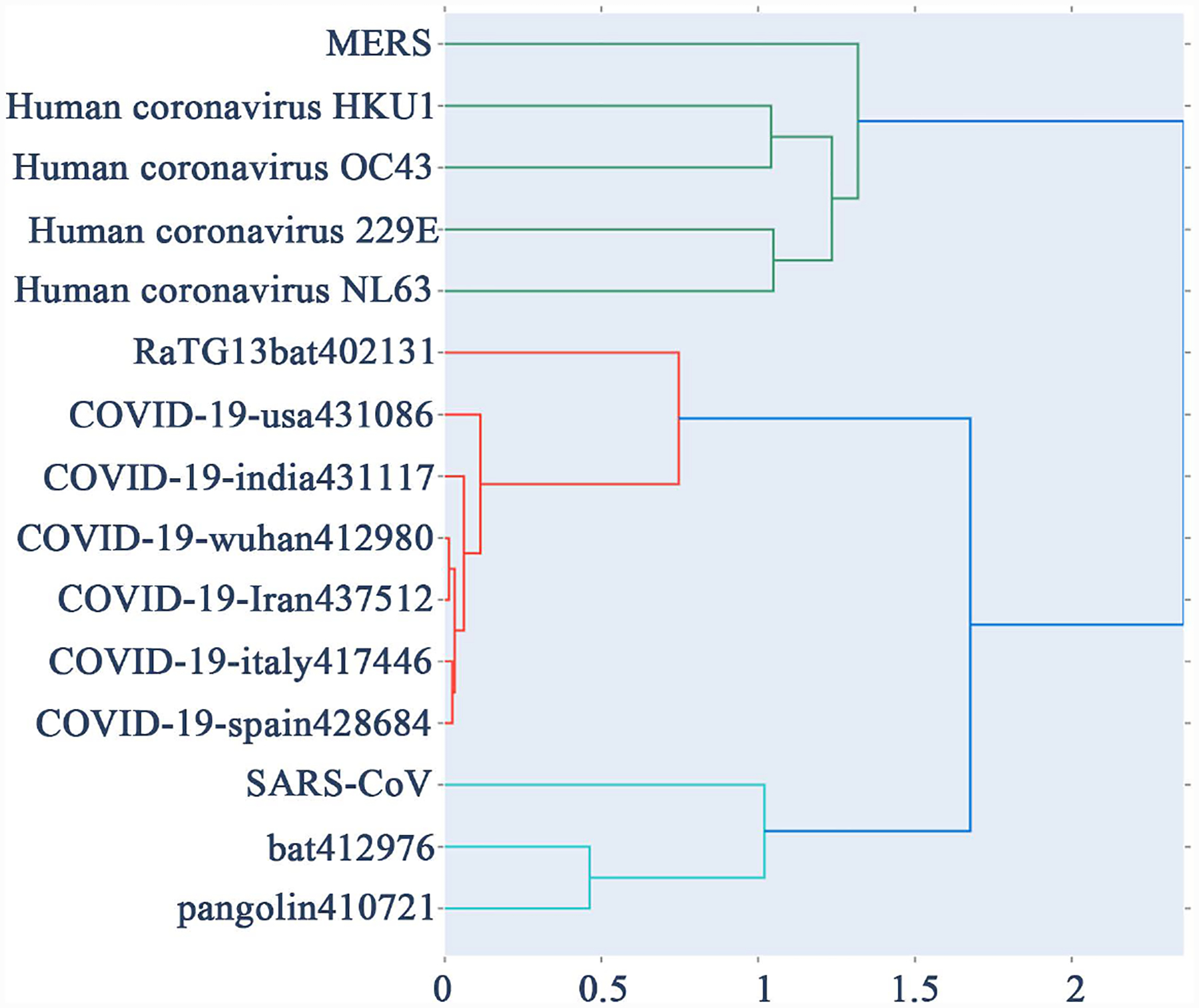
HAC phylogenetic tree using probability matrix distance.

**Figure 3.**
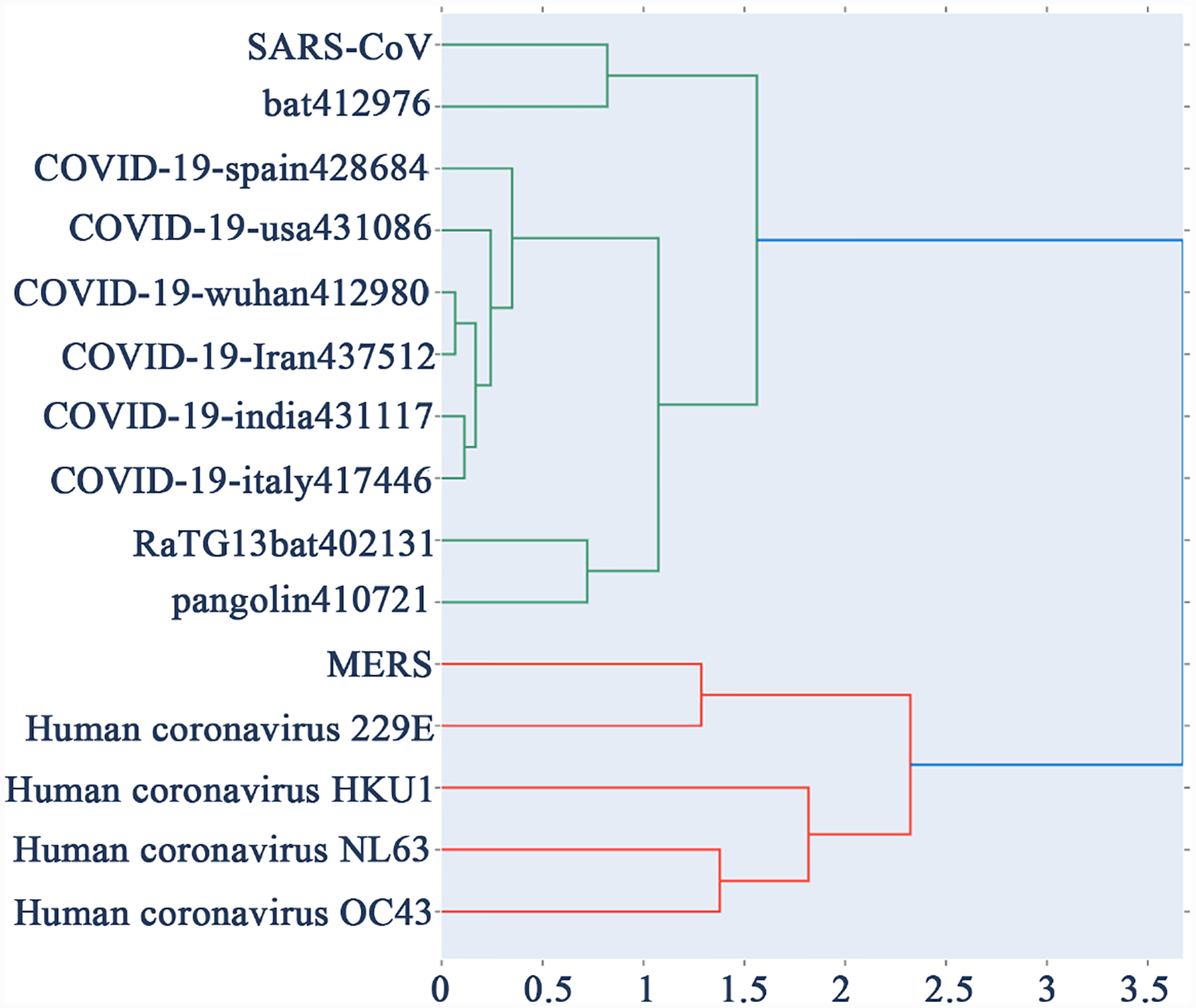
HAC phylogenetic tree using CGR centroid distance.

**Figure 4.**
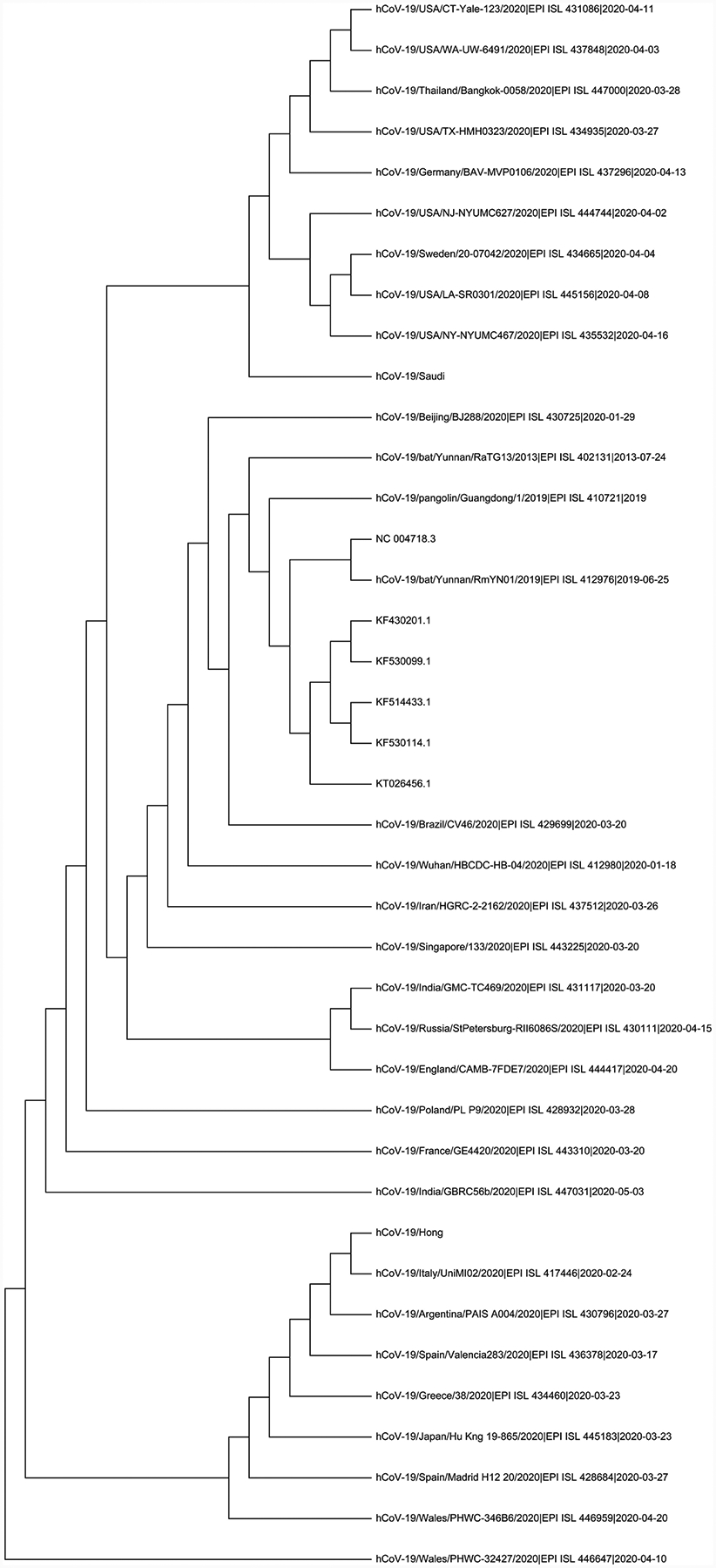
Phylogenetic Tree was created by Clustal X by aligning 15 DNA sequences using Neighborhood Joining Method.

For our dataset we used k = 7, that is, each DNA sequence represented as a 2^7^ × 2^7^ FCGR matrix. In [12], it was found highest accuracy in HIV-1 classification and this value is being used here as it is relevant for our viral analysis. Table 2 display the pairwise distance among 15-virus genomes in the dataset using probability matrix distance while Table 3 display the same using centroid distance. Figure 2 shows the phylogenetic tree obtained using Table 2 distances by python Hierarchical Agglomerative Clustering (HAC) analysis. Similarly Figure 3 shows the phylogenetic tree using Table 3. Figure 4 is the Neighbor Joining Phylogenetic tree using traditional Clustal X method.

From Figure 2 and Figure 4, we can see that the cluster results between Clustal X method and probability distance method are essentially same. Similar Phylogenetic analysis of bat coronaviruses with other coronaviruses and the phylogenetic tree was constructed using Clustal W also done in [15].

**Figure 5.**
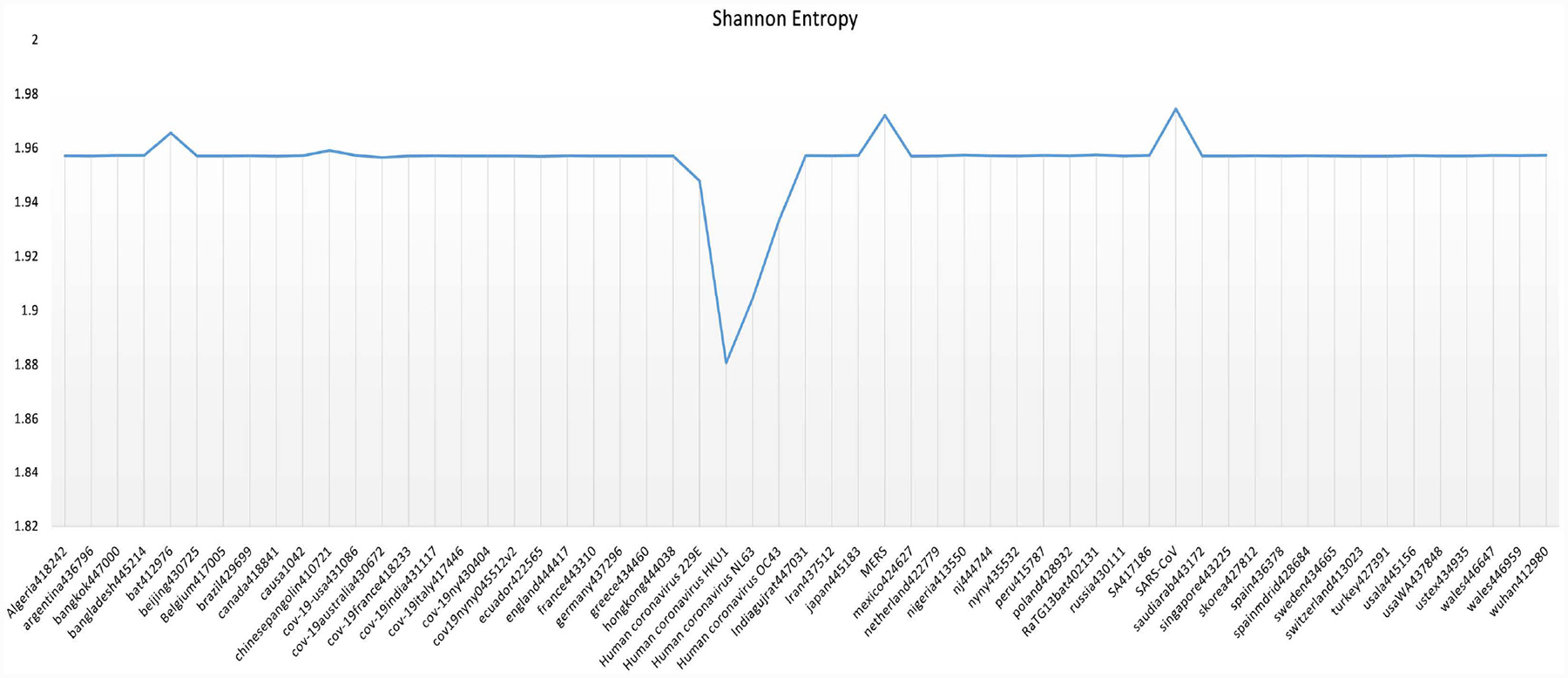
Shannon Entropy of 57-virus genomes.

**Figure 6.**
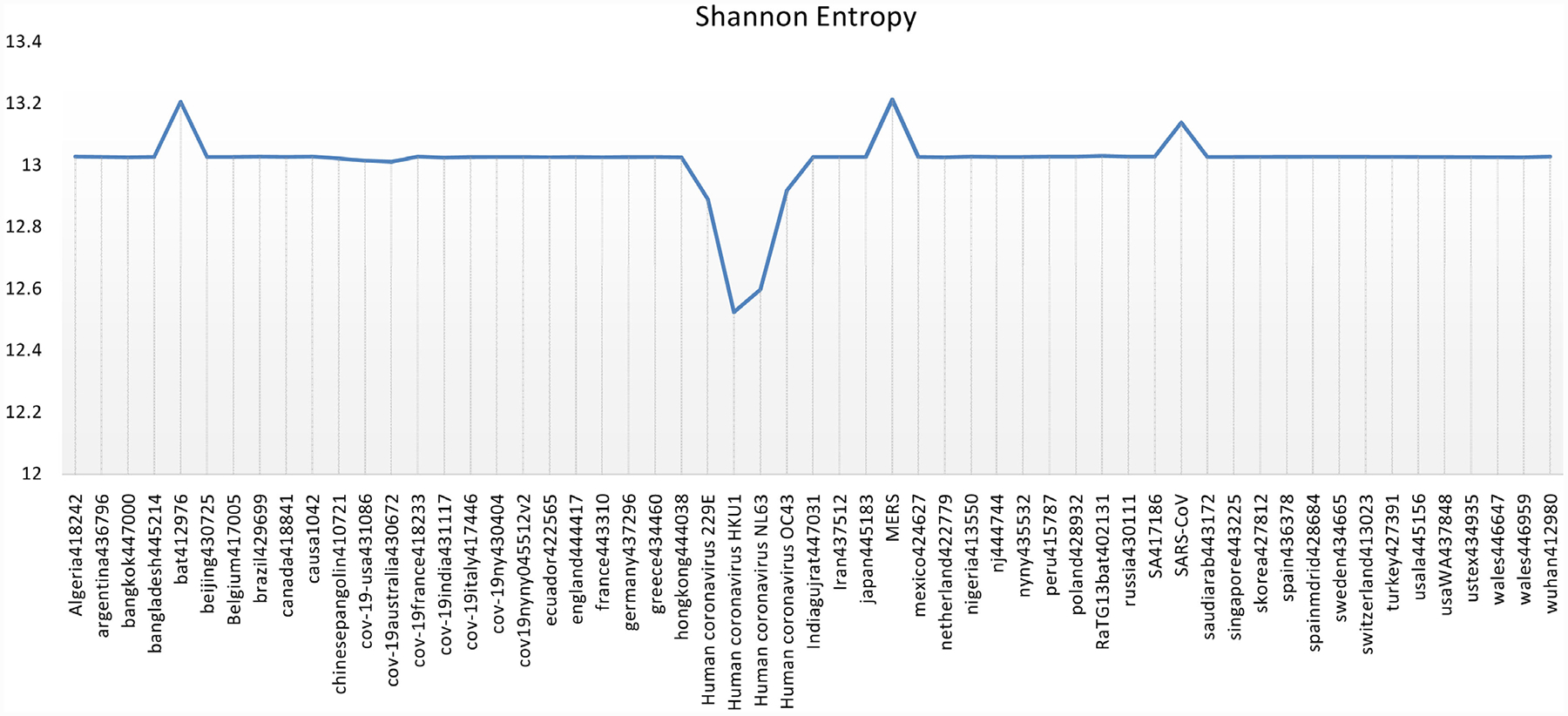
7-mers Shannon Entropy of 57 virus sequences.

All sequence data contain inherent information that can be measured by Shannon’s uncertainty theory. Measuring uncertainty may be used for rapid screening for sequences with matches in available database, prioritizing computational resources, and indicating which sequences with no known similarities are likely to be important for more detailed analysis as seen in [16]. We started with 57 genome sequences and then reduced to 15 based on the Shannon Entropy and Shannon Entropy of 7-mers of the sequences, see Figure 5 and Figure 6. All Covid-19 sequences have entropy close to 1.957. We choose only six Covid-19 sequences in the dataset along with all other sequences with deviated entropy from 1.957 for our analysis of corona viruses.

## 4. Discussion and Conclusions

Our methods are comparable to many other alignment-free methods as shown in [1] [2] [3] [11]. The proposed methods i.e. FCGR Probability and Chaos Centroid, are based on Chaos game representation, which provides a unique and scale-independent representation of DNA sequences through the statistical distribution of *k*-mers along DNA sequences. An advantage of CGR over alignment is that it has the potential to reveal the evolutionary and/or functional relationships between the sequences having no significant homology, as explained in [17]. Furthermore, it does not require prior knowledge of consensus sequences, nor does it involve exhaustive searches for sequences in databases. The limitation of CGR is that it takes a computational time to generate the representations from DNA sequences.

In conclusion, results show that our method can accurately classify different genomic sequences. In terms of classification accuracy, our method is basically the same as the state-of-the art Clustal X and compare with the traditional Clustal X phylogenetic tree construction method [18], our method is much faster. Furthermore, our dendrogram construct method can be widely applicable for various kinds of organisms. This research may contribute to reveal the biological evolution process to some extent, as well as promote the further development of bioinformatics. We may make efforts in our future work to provide a webserver for the methods presented in this paper. All the codes in this paper are written in python and can be available upon request.

## Figures and Tables

**Table 1.** Dataset for the experiment.

Virus name	NCBI/GISAID Accession number
1) hCov-19/bat/Yunnan	EPI_ISL_412976
2) hCov-19/pangolin/Guangdong	EPI_ISL_410721
3) hCov-19/bat/Yunnan/RaTG13	EPI_ISL_402131
4) hCov-19/India	EPI_ISL_431117
5) hCov-19/Italy	EPI_ISL_417446
6) hCov-19/Iran	EPI_ISL_437512
7) hCov-19/Spain	EPI_ISL_428684
8) hCov-19/USA	EPI_ISL_431086
9) hCov-19/Wuhan	EPI_ISL_412980
10) Human Coronavirus-229E	KF-514433
11) Human Coronavirus-HKU1	KF-430201
12) Human Coronavirus-NL63	KF-530114
13) Human Coronavirus-OC43	KF-530099
14) SARS-Cov	NC_004718
15) MERS	KT-026456

**Table 2.** Probability distance matrix of 15 viruses listed in Table 1.

	1	2	3	4	5	6	7	8	9	10	11	12	13	14	15
1															
2	0.3079														
3	0.4900	0.4606													
4	0.5129	0.6301	0.6303												
5	0.7076	0.7548	0.7506	0.7436											
6	0.7342	0.7737	0.7602	0.7969	0.7858										
7	0.8657	0.8700	0.8443	0.9420	0.8850	0.8406									
8	0.8074	0.8299	0.8037	0.8828	0.8587	0.7247	0.7237								
9	0.7578	0.7904	0.7744	0.8132	0.7894	0.7612	0.7067	0.7470							
10	0.4920	0.7671	0.2929	0.6313	0.7441	0.7714	0.8531	0.8123	0.7846						
11	0.4947	0.4750	0.0600	0.6408	0.7608	0.7614	0.8519	0.8029	0.7827	0.3143					
12	0.4930	0.4677	0.0321	0.6341	0.7553	0.7602	0.8477	0.8028	0.7783	0.3024	0.0299				
13	0.4905	0.4644	0.0180	0.6311	0.7529	0.7601	0.8456	0.8032	0.7757	0.2972	0.0492	0.0200			
14	0.4901	0.4646	0.0179	0.6318	0.7524	0.7595	0.8451	0.8030	0.7748	0.2978	0.0530	0.0254	0.0168		
15	0.4907	0.4623	0.0095	0.6306	0.7514	0.7599	0.8444	0.8037	0.7748	0.2953	0.0583	0.0320	0.0192	0.0192	

**Table 3.** CGR Centroid distance matrix of 15 viruses listed in Table 1.

	1	2	3	4	5	6	7	8	9	10	11	12	13	14	15
1															
2	0.4531														
3	0.5567	0.4439													
4	0.5408	0.6281	0.6188												
5	0.9029	0.9255	0.8784	0.7598											
6	0.8845	0.8718	0.8409	0.8615	0.8762										
7	1.4297	1.3203	1.2682	1.3924	1.300	1.2339									
8	1.2246	1.0924	1.0161	1.2011	1.200	0.9157	0.9635								
9	1.0256	0.9862	0.9295	0.9869	0.9310	0.8623	0.9538	0.9123							
10	0.5581	0.4575	0.3303	0.6356	0.9271	0.8824	1.2759	1.0163	0.9912						
11	0.5915	0.4816	0.1350	0.6525	0.9115	0.8667	1.2682	1.0432	0.9391	0.3694					
12	0.5654	0.4591	0.0969	0.6312	0.8839	0.8518	1.2604	1.0403	0.9217	0.3446	0.0670				
13	0.5607	0.4576	0.0702	0.6247	0.8837	0.8450	1.2644	1.0326	0.9291	0.3367	0.1156	0.0636			
14	0.6113	0.5127	0.1596	0.6785	0.9097	0.8583	1.3064	1.0584	0.9613	0.3859	0.2254	0.1793	0.1558		
15	0.5460	0.4416	0.0454	0.6167	0.8783	0.8332	1.2680	1.0221	0.9235	0.3290	0.1295	0.0943	0.0721	0.1586	

## References

[1] Ni HM, Qi DW and Mu H (2018) Applying MSSIM Combined Chaos Game Representation to Genome Sequences Analysis. Genomics, 110, 180–190. 10.1016/j.ygeno.2017.09.010 28941638

[2] Stan C, Cristescu CP and Scarlat EI (2010) Similarity Analysis for DNA Sequences Based on Chaos Game Representation. Case Study: The Albumin. Journal of Theoretical Biology, 267, 513–518. 10.1016/j.jtbi.2010.09.027 20869369

[3] Li Y, He L, (2017) A Novel Fast Vector Method for Genetic Sequence Comparison. Scientific Reports, 7, Article No. 12226. 10.1038/s41598-017-12493-2 PMC5610321 28939913

[4] Jeffrey HJ (1990) Chaos Game Representation of Gene Structure. Nucleic Acids Research, 18, 2163–2170. 10.1093/nar/18.8.2163 2336393 PMC330698

[5] Goldman N (1993) Nucleotide, Dinucleotide and Trinucleotide Frequencies Explain Patterns Observed in Chaos Game Representations of DNA Sequences. Nucleic Acids Research, 21, 2487–2491. 10.1093/nar/21.10.2487 8506142 PMC309551

[6] Kari L, Hill KA, Sayem AS, Karamichalis R, Bryans N, (2015) Mapping the Space of Genomic Signatures. PLoS ONE, 10, e0119815 10.1371/journal.pone.0119815 26000734 PMC4441465

[7] Almeida JS, Carriço JA, Maretzek A, Noble PA and Fletcher M (2001) Analysis of Genomic Sequences by Chaos Game Representation. Bioinformatics, 17, 429–437. 10.1093/bioinformatics/17.5.429 11331237

[8] Wang Y, Hill K, Singh S and Kari L (2005) The Spectrum of Genomic Signatures: From Di-Nucleotides to Chaos Game Representation. Gene, 346, 173–185. 10.1016/j.gene.2004.10.021 15716010

[9] Deschavanne P, Giron A, Vilain J, Fagot G and Fertil B (1999) Genomic Signature: Characterization and Classification of Species Assessed by Chaos Game Representation of Sequences. Molecular Biology and Evolution, 16, 1391–1399. 10.1093/oxfordjournals.molbev.a026048 10563018

[10] Karamichalis R, Kari L, Konstantinidis S, (2015) An Investigation into Inter- and Intra-Genomic Variations of Graphic Genomic Signatures. BMC Bioinformatics, 16, Article No. 246. 10.1186/s12859-015-0655-4 PMC4527362 26249837

[11] Tanchotsrinon W, Lursinsap C and Poovorawan Y (2015) A High Performance Prediction of HPV Genotypes by Chaos Game Representation and Singular Value Decomposition. BMC Bioinformatics, 16, 71 10.1186/s12859-015-0493-4 25880169 PMC4375884

[12] Solis-Reyes S, Avino M and Poon A (2018) An Open-Source k-mer Based Machine Learning Tool for Fast and Accurate Subtyping of HIV-1 Genomes. PLoS ONE, 13, e0206409 10.1371/journal.pone.0206409 30427878 PMC6235296

[13] WHO (2019) Middle East Respiratory Syndrome Coronavirus (MERS-CoV). http://www.who.int/emergencies/mers-cov/en

[14] WHO (2020) Summary Table of SARS Cases by Country, November 1, 2002-August 7, 2003. http://www.who.int/csr/sars/country/2003_08_15/en

[15] Hu B, Ge X, Wang L, (2015) Bat Origin of Human Coronaviruses. Virology Journal, 12, 221 10.1186/s12985-015-0422-1 26689940 PMC4687304

[16] Akhter S, Bailey B, Salamon P, (2013) Applying Shannon’s Information Theory to Bacterial and Phage Genomes and Metagenomes. Scientific Reports, 3, Article No. 1033. 10.1038/srep01033 PMC3539204 23301154

[17] Basu S, Pan A, Dutta C and Das J (1997) Chaos Game Representation of Proteins. Journal of Molecular Graphics and Modelling, 15, 279–289. 10.1016/S1093-3263(97)00106-X 9640559

[18] Larkin MA, (2007) Clustal W and Clustal X Version 2.0. Bioinformatics, 23, 2947–2948. 10.1093/bioinformatics/btm404 17846036

